# Working Women in Large Towns

**Published:** 1890-08-16

**Authors:** 


### Working Women in Large Towns.

### VII.—MATCHBOX, BRUSH, AND BOX MAKERS.

### Matchbox Makers.

Matchbox making is an industry largely resorted to by home-workers in certain districts of the East-end, notably in Shoreditch and Bethnal Green. Climb the steep dirty staircase of house after house in some of the smaller streets, and you will find the rooms strewn with matchboxes laid out to dry, and one or more workers at the small table, busily engaged in dividing up thin slips of wood, laying them upon coloured paper, wet with paste, and then binding the boxes into shape with what looks to the outsider remarkable dexterity. Not that the work really requires much skill, for even a small child can make matchboxes, and if quick, can earn a not inconsiderable wage (as things go), which must be a great temptation to the mother to keep it from school. Nevertheless, there is a good deal of variation in the respective "out-puts" of work among the matchbox makers. It appears that exceptionally good and industrious workers can clear one-and-threepence or possibly one and sixpence a day, when in full employ; but the great majority do not earn so much. Some of them, it is thought, do not really do their best, and prefer charitable assistance to hard work, merely doing a certain amount in order to keep up an appearance of industry. But while this may be the case with some, there are others who work away in grim earnest, and who are greatly to be pitied for the dismal disproportion between their labour and their pay. Some of them have no other means of subsistence; others have husbands who are ill, or constantly out of work; in either case, the rooms are wretchedly furnished, the food scanty, and the work, though comparatively light, one long grind. Here are three cases come upon in succession by one who was visiting the matchbox makers for quite another purpose than that of discovering pathetic cases.

In the first house was a young woman with a pale weary face, whose husband was then in hospital. He could never do much, being very asthmatical; and she herself had been too poorly to touch her work the day before. There were five children, four at school, the other playing among the heap of matchboxes which strewed the floor. As she talked, she went on mechanically with her work—covering the match-box trays with paper of a bright and rather artistic red, and slipping in the little wooden bottoms for them. She was paid at the usual rate (for small boxes) of twopence farthing per gross, and by working hard all day could earn about one-and-threepence, having to spend a penny or two in finding paste and hemp for tying up the bundles of boxes.

In the next house the worker was a young widow, who could hardly have been more than three-and-twenty, but had five children. The room was small and dirty, and the bed as well as the floor strewn with boxes, for which, being the larger kind, she was paid twopence-halfpenny per gross. She had learnt her trade early, and seemed to be an unusually quick worker; but with five children to look after, it would be impossible to work very long hours. The eldest little girl was a useful help to her out of school-hours, but could not make the boxes throughout, for, as the mother said, putting the rough paper on the sides made her fingers bleed.

In the third house was the family of a "docker" who had been out of work from illness for several weeks. A daughter of twenty worked at a factory; a younger girl was learning fancy-box making, and received but a shilling a-week; and the eldest daughter, a hopeless cripple, worked with her mother at matchboxes, the mother making the box, with its picture of General Gordon on the outside, the daughter slowly labouring away at the trays, which she was covering with bright yellow paper. The poor girl was evidently "not quite bright," as well as terribly crippled; she never left the room in which she worked all day, being too bad even to use crutches, though they had done all they could for her at a children's hospital; yet she seemed bright and contented, and evidently did her little best to help mother. We think it probable that, in the two first cases, charitable assistance was given—indeed, it is difficult to see how the poor workers could possibly live without it—but the last-mentioned woman was of a sturdy, independent kind, though evidently very poor; and she seemed to look to efforts on the part of the workers themselves, rather than to charity, as a remedy for their troubles. Efforts are now being made to form a Matchbox Makers' Union; and although we are not very sanguine as to its success, it must certainly have the good wishes of all who sympathise with the poor. Foreign competition is already keenly felt in the trade, there being whole roomfuls of Swedish boxes even now in some of the match factories; and it may be that matchbox making is doomed—so far at least as England is concerned. Still, there may be yet some opportunity for improving the position of the poor workers; and even if wages cannot be raised, that is by no means the only use of a Union, as the world cannot too often be reminded.

### Box Makers.

Box-making is nearly always learnt in a factory, and fancy-boxes are but rarely given to out-door workers; but a large number of plain boxes, for buttons, boots, soap, butter-scotch, wedding-cake, &c., are made by home-workers. The fact that they have once been factory hands, and are therefore in touch with the indoor workers, no doubt tends to prevent the playing off of one set of workers against the other, and thus helps to keep up the rate of wages; and some box-makers do well, earning from 1s. 8d. to 2s. a day. But the manufacturers of the smaller and cheaper class of boxes themselves confess that they sell their goods at a rate which could not be remunerative if they had to provide accommodation for their workers. By giving the work out, however, they throw the cost of room, firing, gas, &c., upon the worker, and, as the conjurors say—that's how it's done. All box-making is, to some extent, skilled labour, and, therefore, it is not so badly paid as some industries; but we fear that where home-workers have to support themselves entirely, they are generally in sorely straitened circumstances. Not only are their wages low, but the work is irregular; and it must be remembered that in this, as in all home industries, much time is lost in fetching and carrying back the work—not to speak of the money which has to be expended on tram-fares, when the worker lives at some distance from the manufactory.

### Brush Makers.

Brush-making again is generally learnt in a factory. The heavy goods are always done indoors, but tooth-brushes, boot-brushes, scrubbing and stove brushes, etc., are largely given out. Women who work for the large factories seem to earn good wages, for only those who are known to the employers as skilful and honest are allowed to take work home. Miss Collet mentions a mother and two daughters, engaged in tooth-brush making, who stated that their joint earnings amounted to twenty-five shillings a-week; and as they probably worked only nine hours a day, they were evidently in fairly comfortable circumstances. But a good deal of brush-making is done by women who work for a man employing only two or three hands. These brush-makers are by no means so fortunate. They are paid at a much lower rate, and seldom have constant work. Sometimes they are helped by a father or brother, who can turn the backs and handles, and sell the brushes when done; but here again the work is irregular. We are told that many of these home-working brush-makers try to keep up their strength and spirits by dram-drinking; but when we remember that the work is fatiguing, as well as uncertain and badly paid, we can scarcely find it in our hearts to think very badly of them for this.

*(To be continued.)*

